# Measurement of Acne Severity, Dietary Habits, and Blood Zonulin Levels in Acne Patients

**DOI:** 10.1111/jocd.70083

**Published:** 2025-02-27

**Authors:** Rahime Cankat Gürel, Mehmet Yıldırım, İjlal Erturan, Selma Korkmaz, Duygu Kumbul Doğuç

**Affiliations:** ^1^ Department of Dermatology, Faculty of Medicine Suleyman Demirel University Faculty of Medicine Isparta Turkey; ^2^ Department of Biochemistry, Faculty of Medicine Suleyman Demirel University Isparta Turkey

**Keywords:** acne, diet, intestinal permeability, zonulin

## Abstract

**Background:**

Zonulin is a signaling protein secreted by enterocytes that can reversibly open tight junctions. Blood zonulin levels are elevated in disorders characterized by increased intestinal permeability.

**Aims:**

The aim of this study is to compare blood zonulin levels and dietary habits between acne vulgaris patients and healthy controls, as well as to investigate the relationship between blood zonulin levels, diet, disease severity, age at onset, and duration in the acne group. Additionally, we aimed to explore the relationship between diet and blood zonulin levels in both groups.

**Patients/Methods:**

Seventy‐five patients with acne vulgaris over the age of 18 years and 75 healthy controls were included in the study. Blood zonulin levels in both groups were analyzed using the sandwich ELISA method. Demographic data, habits, and dietary preferences were recorded.

**Results:**

In the acne group, the mean blood zonulin level was significantly higher than in the control group (195.54 ± 49.05 ng/mL vs. 158.45 ± 85.07 ng/mL, *p* = 0.001). Additionally, milk and high glycemic index food consumption were significantly higher in the acne group.

**Conclusions:**

Our results support the hypothesis that increased intestinal permeability may be involved in the pathogenesis of acne vulgaris. Moreover, our study confirms the relationship between diet and acne. Future studies are warranted to elucidate the impact of acne‐promoting and acne‐preventive foods on the pathogenesis of acne through intestinal permeability.

## 1 Introduction

Acne vulgaris is a multifactorial chronic inflammatory disease of the pilosebaceous unit. Although the etiology of acne vulgaris is not fully understood, it is postulated that genetic, immune, hormonal, and environmental factors, including nutritional influences, may collectively contribute to its pathogenesis [1]. In the literature on the relationship between acne and diet, there is a consistent association between the consumption of foods with a high glycemic index and dairy‐containing foods and the development of acne. The concept of increased intestinal permeability as a consequence of dysbiosis in the pathogenesis of acne vulgaris was initially proposed by dermatologists John H. Stokes and Donald M. Pillsbury in the 1930s. Despite having been previously dismissed, recent evidence has emerged suggesting that increased intestinal permeability may play a role in the pathogenesis of acne [2]. Zonulin is a signaling protein that reversibly opens tight junctions between intestinal epithelial cells, thereby increasing intestinal permeability [3].

The objective of our study was to examine the relationship between zonulin and dietary habits, with a particular focus on the role of diet in acne pathogenesis through intestinal permeability. This is the first study in the literature to examine the relationship between acne vulgaris and intestinal permeability through dietary habits and blood zonulin levels.

## 2 Materials and Methods

Patients over 18 years old with acne vulgaris and healthy controls matched in terms of age and gender who applied to the dermatology outpatient clinic of our hospital between October 2022 and March 2023 were included in the study. All participants were volunteers and provided informed consent. Our study was approved by the ethics committee of our hospital on October 27, 2022. Patients with systemic diseases, including celiac disease, Type 1 diabetes mellitus, autoimmune thyroid disease, and inflammatory bowel disease, were excluded. Furthermore, individuals with hyperandrogenism (including those with polycystic ovary syndrome, androgenetic alopecia, and hirsutism) were excluded from the acne group.

Following a 12‐h fast, 10 mL of venous blood was collected from each participant for the assessment of zonulin levels and placed in gel vacuum biochemistry tubes. Following a coagulation period of 10–20 min at room temperature, the serum was separated by centrifugation at 4000 rpm for a period of five minutes. The sera were subsequently transferred to Eppendorf tubes and stored at −80°C. Serum zonulin levels were quantified using a commercial sandwich ELISA (enzyme‐linked immunosorbent assay) kit (Elabscience, No. E‐EL‐H5560, 14 780 Memorial Drive, Suite 216, Houston, Texas 77 079). The sensitivity of the kit was 0.47 ng/mL.

The severity of acne was assessed using the Global Acne Grading Scale (GAGS) for patients with acne vulgaris. The type of acne, the duration of the disease, and whether the patients in the acne group received treatment for acne were identified. Furthermore, the two groups were queried regarding additional demographic data, including height, weight, body mass index (BMI), exercise habits, and sleep duration.

A questionnaire was utilized to analyze the dietary habits of both groups. The dietary questionnaire inquired about the consumption of various food items, including milk, dairy products, lactose‐containing foods, eggs, red meat, white meat, fish, vegetables, fruits, cereals, legumes, olive oil, solid oils, nuts, fast foods, ready‐to‐eat desserts, soft drinks, and chocolate. The quantity of each food item consumed was calculated in grams and milliliters.

### 2.1 Statistical Analysis

These data were analyzed using the Statistical Package for the Social Sciences (SPSS) version 22. Descriptive statistics were presented as the mean ± standard deviation. Pearson chi‐squared test was used for categorical variables. The suitability of continuous variables for normal distribution was assessed using the Kolmogorov–Smirnov and Shapiro–Wilk tests. Continuous variables that do not have a normal distribution were assessed using the Mann–Whitney *U* and Kruskal–Wallis tests. Conversely, continuous variables which have a normal distribution were assessed using independent samples *t* test and ANOVA tests. Spearman's rank correlation coefficient was used for correlation analysis. The variables were analyzed at the 95% confidence interval, and a *p* value of less than 0.05 was considered statistically significant.

## 3 Results

A total of 150 patients were included in our study, with 75 assigned to the acne group and 75 to the control group. No statistically significant differences were observed between the demographic characteristics, habits, sleep durations, or exercise frequencies of the two groups (Table 1).

**TABLE 1:** Demographic data and body mass indexes of the acne and control groups.

Demographic data and body mass index	Acne group (*n*:75) Mean ± sd	Control group (*n*:75) Mean ± sd	*p*
Age	23.19 ± 3.83	23.57 ± 3.09	0.498*
Body mass index (kg/m^2^)	21.63 ± 3.20	22.58 ± 3.66	0.129**

	*n* (%)	*n* (%)	
Sex			1
Female	43 (57.3)	43 (57.3)	
Male	32 (42.7)	32 (42.7)	

*Note:* The Pearson chi‐squared test was used to compare categorical variables, *Independent samples *t*‐test, **Mann–Whitney *U*‐test, *p* < 0.05^a^. The symbol “a” indicates that the calculated *p*‐value is statistically significant.

Abbreviations: kg, kilogram; m, meter; m^2^, square meter; sd, standard deviation.

The mean disease duration of the patients in the acne group was 68.16 ± 48.28 months. Among the patients, 66 (88%) had adolescent acne, whereas 9 (12%) had postadolescent acne (*p* = 0.001). There were 11 patients (14.7%) with mild acne, 41 patients (54.7%) with moderate acne, and 23 patients (30.7%) with severe acne (*p* = 0.001). No significant relationships were detected between the severity of acne and the following factors: smoking habits, alcohol consumption, exercise routines, duration of sleep, mean weight, and onset age of acne.

A comparison of the zonulin levels between the two groups revealed that the patient group had a significantly higher mean blood zonulin level (195.54 ± 49.05) than the control group (158.45 ± 85.07) (*p* = 0.001). No significant relationship was detected between blood zonulin levels and acne severity. A significant correlation was observed between body mass index and blood zonulin levels in the acne group (*r* = 0.309, *p* = 0.007). In patients with acne, the mean blood zonulin level was significantly higher in alcohol users than in nonusers (*p* = 0.026). Similarly, the mean blood zonulin level was significantly higher in males with acne than in females with acne (*p* = 0.009).

A comparison of consumption preferences according to dietary habits in both groups is presented in Table 2. A comparative analysis of the amount of food consumed by the acne and control groups is presented in Table 3. A significant relationship was identified between the consumption of legumes and the severity of acne. Individuals with severe acne exhibited a notable preference for legumes (*p* = 0.038). No significant relationship was detected between the mean consumption of other foods and acne severity.

**TABLE 2:** Consumption preferences of various foods in the acne and control groups.

Foods	Acne group *n* (%)	Control group *n* (%)	*p*
Milk	**50** (**66**.**7**)	**34** (**45**.**3**)	**0**.**008** ^a^
> 1 glass/day milk	**14** (**18**.**7**)	**2** (**2**.**7**)	**0**.**002** ^a^
Whole milk	**19** (**38**)	**20** (**60**.**6**)	**0**.**043** ^a^
Semi‐skimmed milk	**31** (**62**)	**13** (**39**.**4**)	
Dairy products	65 (86.7)	71 (94.7)	0.092
> 1 glass/day dairy product	41 (54.7)	30 (40)	0.072
> 1 portion/day fast food	**22** (**29**.**3**)	**8** (**10**.**7**)	**0**.**004** ^a^
Ready‐to‐eat‐dessert	**53** (**70**.**7**)	**39** (**52**)	**0**.**019** ^a^
> 2 glasses/day sugary soft drink	**12** (**16**)	**4** (**5**.**3**)	**0**.**034** ^a^
Chocolate	61 (81.3)	60 (80)	0.836
Milk chocolate	**53** (**86**.**9**)	**38** (**63**.**3**)	**0**.**003** ^a^
Dark chocolate	**8** (**13**.**1**)	**22** (**36**.**7**)	
> 1 tablespoon/day olive oil	**11** (**37**.**9**)	**46** (**62**.**2**)	**0**.**026** ^a^
Fish	**19** (**25**.**3**)	**55** (**73**.**3**)	**0**.**0001** ^a^
Red meat	**73** (**97**.**3**)	**56** (**74**.**7**)	**0**.**0001** ^a^
> 1 portion/week red meat	**46** (**61**.**3**)	**26** (**34**.**7**)	**0**.**001** ^a^
White meat	69 (92)	70 (93.3)	0.754
> 1 portion/week white meat	53 (70.7)	54 (72)	0.857
> 2 portions/day processed meat	**15** (**20**)	**4** (**5**.**3**)	0.007^a^
> 1 portion/day vegetables	33 (44)	28 (37.3)	0.406
Fruits	**28** (**37**.**3**)	**59** (**78**.**7**)	**0.0001** ^a^
Egg	**48** (**64**)	**61** (**81**.**3**)	**0.017** ^a^
> 1 piece/day egg	**13** (**17**.**3**)	**28** (**37**.**3**)	**0.006** ^a^
> 1 piece/day refined cereals	**54** (**72**)	**31** (**41**.**3**)	**0.0001** ^a^
> 1 portion/week legumes	**64** (**85**.**3**)	**53** (**70**.**7**)	**0.030** ^a^
Nuts	**43** (**57**.**3**)	**57** (**76**)	**0.015** ^a^
> 1 portion/day nuts	**33** (**44**)	**45** (**60**)	**0.05** ^a^
Solid fat	57 (76)	54 (72)	0.577

*Note:* Pearson chi‐squared test was used, *n*: number of people, *p* < 0.05^a^, 1 portion = 100 g, 1 glass = 200 milliliters, 1 tablespoon = 15 milliliters, 1 portion processed meat or nuts = 40 g. The symbol “a” indicates that the calculated *p*‐value is statistically significant.

**TABLE 3:** Food consumption amounts of the acne and control groups.

Foods	Acne group Mean ± sd	Control group Mean ± sd	*p*
Red meat (gr)	**218**.**00 ± 192**.**36**	**108**.**67 ± 82**.**76**	**0**.**0001** ^a^ **
White meat (gr)	291.60 ± 415.24	268.67 ± 296.69	0.966**
Fish (gr)	**25**.**33 ± 48**.**88**	**98**.**07 ± 78**.**97**	**0**.**0001** ^a^ **
Vegetables (gr)	150.73 ± 120.11	156.20 ± 116.96	0.708**
Fruits (gr)	**61**.**87 ± 118**.**85**	**94**.**93 ± 76**.**97**	**0**.**0001** ^a^ **
Milk (ml)	**142**.**27 ± 146**.**26**	**62**.**07 ± 96**.**32**	**0**.**0001** ^a^ **
Dairy products (ml)	251.93 ± 215.,53	202.67 ± 124.86	0.158**
Lactose‐containing foods (gr)	**371**.**53 ± 285**.**34**	**265**.**27 ± 166**.**04**	**0**.**021** ^a^ **
Egg (piece)	**0**.**83 ± 1**.**03**	**1**.**24 ± 0**.**93**	**0**.**001** **
Sugary soft drink (ml)	229.00 ± 297.75	156.87 ± 186.91	0.330**
Chocolate (gr)	21.81 ± 24.06	17.13 ± 19.90	0.366**
Dark chocolate (gr)	9.63 ± 4.87	20.27 ± 21.01	0.159**
Milk chocolate (gr)	29.42 ± 24.71	22.08 ± 19.78	0.169**
Fast food (gr)	104.53 ± 105.92	72.33 ± 93.21	0.119**
Ready‐to‐eat dessert (gr)	**184**.**00 ± 189**.**08**	**118**.**00 ± 150**.**36**	**0**.**0001** ^a^ **
Refined cereals (gr)	**204**.**40 ± 121**.**25**	**143**.**40 ± 89**.**01**	**0**.**0001** ^a^ **
Legumes (gr)	**306**.**00 ± 196**.**24**	**242**.**00 ± 161**.**71**	**0**.**019** ^a^ **
Processed meat (gr)	**48**.**93 ± 72**.**22**	**25**.**60 ± 37**.**81**	**0**.**040** ^a^ **
Nuts (gr)	**47**.**93 ± 58**.**24**	**78**.**80 ± 89**.**66**	**0**.**015** ^a^ **
Olive oil (ml)	**7**.**33 ± 13**.**28**	**32**.**00 ± 21**.**85**	**0**.**0001** ^a^ **
Solid oil (gr)	9.81 ± 9.88	7.91 ± 8.14	0.276**

*Note:* The symbol “a” indicates that the calculated *p*‐value is statistically significant.

Abbreviations: gr, gram; ml, milliliter; sd, standard deviation.

** Mann–Whitney *U*‐test, *p* < 0.05^a^.

Table 4 presents the mean blood zonulin levels of the patients and controls included in the study with respect to their dietary intake.

**TABLE 4:** Blood zonulin levels according to food consumption.

	Acne zonulin Mean ± sd	Control zonulin Mean ± sd	*p*
Food consumption	195.54 ± 49.05	158.45 ± 85.07	0.001^a^ **
Milk consumers	201.58 ± 42.94	182.46 ± 85.08	0.233*
More than 1 glass/day milk consumers	193.87 ± 36.60	198.80 ± 89.44	0.874**
Semi‐skimmed milk consumers	206.19 ± 41.69	212.61 ± 95.97	0.820*
Whole milk consumers	194.05 ± 45.00	159.81 ± 73.47	0.090*
More than1 glass/day dairy products consumers	200.35 ± 47.77	173.61 ± 87.11	0.135*
More than1 glass/day lactose‐containing food consumers	199.79 ± 46.39	174.76 ± 79.63	0.052**
Fast food consumers	**194**.**74 ± 48**.**87**	**148**.**65 ± 77**.**53**	**0**.**0001** ^a^ *
Ready‐to‐eat desserts consumers	**196**.**29 ± 47**.**75**	**115**.**46 ± 55**.**38**	**0**.**001** ^a^ **
Sugary soft drink consumers	**199**.**76 ± 48**.**04**	**165**.**21 ± 75**.**35**	**0**.**011** ^a^ *
Chocolate consumers	**191**.**65 ± 50**.**54**	**149**.**00 ± 81**.**10**	**0**.**001** ^a^ *
Dark chocolate consumers	172.28 ± 57.74	176.17 ± 79.59	0.901*
Milk chocolate consumers	**194**.**58 ± 49**.**31**	**133**.**28 ± 78**.**74**	**0**.**0001** ^a^ *
Olive oil consumers	152.15 ± 37.18	158.44 ± 85.64	0.930**
More than 1 tablespoon/day olive oil consumers	126.88 ± 13.88	123.96 ± 65.54	0.762**
Fish consumers	190.83 ± 42.64	185.90 ± 71.62	0.778*
Red meat consumers	**194**.**87 ± 49**.**23**	**165**.**25 ± 86**.**67**	**0**.**020** ^a^ **
More than 1 portion/week red meat consumers	204.90 ± 41.15	175.37 ± 98.95	0.157*
White meat consumers	**196**.**44 ± 48**.**48**	**159**.**73 ± 86**.**86**	**0**.**003** ^a^ *
More than 1 portion/week white meat consumers	**197**.**20 ± 46**.**76**	**164**.**15 ± 87**.**99**	**0**.**009** ^a^ **
More than 2 portions/week white meat consumers	**210**.**92 ± 45**.**28**	**170**.**14 ± 91**.**48**	**0**.**041** ^a^ *
Processed meat consumers	**199**.**00 ± 44**.**96**	**166**.**05 ± 81**.**00**	**0**.**033** ^a^ *
More than 1 portion/day vegetable consumers	194.56 ± 50.65	165.19 ± 91.50	0.138*
Fruit consumers	170.36 ± 43.42	143.18 ± 73.61	0.360**
Egg consumers	**196**.**18 ± 48**.**68**	**159**.**72 ± 88**.**01**	**0**.**006** ^a^ **
More than 1 portion/day refined cereals consumers	199.09 ± 47.94	171.29 ± 99.23	0.137**
More than 2 portions/day refined cereals consumers	204.63 ± 52.65	189.01 ± 106v90	0.654*
More than 1 portion/week legume consumers	**194**.**41 ± 49**.**55**	**161**.**85 ± 89**.**61**	**0**.**021** ^a^ *
Nuts consumers	**195**.**68 ± 49**.**65**	**154**.**88 ± 85**.**47**	**0**.**004** ^a^ *
More than 1 portion/day nuts consumers	**190**.**59 ± 49**.**50**	**144**.**50 ± 79**.**97**	**0**.**002** ^a^ *
Solid oil consumers	**202**.**95 ± 46**.**39**	**154**.**02 ± 84**.**05**	**0**.**0001** ^a^ *

*Note:* The symbol “a” indicates that the calculated *p*‐value is statistically significant.

* Independent samples *t*‐test.

** Mann–Whitney *U*‐test, sd: standard deviation, *p* < 0,05^a^, 1 portion = 100 g, 1 glass = 200 mL, 1 tablespoon = 15 mL, 1 portion of processed meat or nuts = 40 g.

Correlation analysis revealed a positive correlation between blood zonulin levels and red meat consumption in the acne group (*r* = 0.273, *p* = 0.018). No significant correlation was detected between zonulin and other foods. Additionally, no significant correlations were detected between the onset age of acne, duration of disease, zonulin level, sleep duration, and the acne quality‐of‐life index.

## 4 Discussion

Zonulin is a protein that is released from enterocytes in response to enterotoxins (bacterial toxins, gluten etc.) and increases intestinal permeability by reversibly opening tight junctions. Elevated blood zonulin levels and impaired intestinal permeability have been documented in numerous systemic inflammatory disorders [3]. In our study, the mean blood zonulin level, which serves as a reflection of increased intestinal permeability, was found to be significantly higher in the acne group compared to the control group (*p* = 0.001). However, no significant correlation was observed between mean zonulin levels and acne severity (*p* = 0.191). These findings indicated an elevated intestinal permeability in individuals with acne, which was corroborated by the literature on blood zonulin levels in other inflammatory dermatological conditions, such as vitiligo and rosacea [4, 5]. The mean blood zonulin level was found to be significantly higher in patients with acne who used alcohol than in those who did not (*p* = 0.026). As documented in the literature, alcohol consumption has been linked to increased intestinal permeability, which can be attributed to alterations in the composition of the intestinal microbiome [3].

A number of studies that demonstrate a correlation between diet and acne have recently been conducted. A significant body of literature indicates a relationship between milk consumption and acne. The association between milk and acne has been attributed to the presence of a growth hormone, insulin‐like growth factor 1 (IGF‐1), which is believed to be more prevalent in milk with a reduced fat content [6]. The mean amount of milk consumed (*p* = 0.0001) and the number of individuals consuming semi‐skimmed milk were found to be higher in the acne group (*p* = 0.043) in our study. In a multicenter study, the number of individuals consuming milk and the amount of milk consumed were significantly higher in those with acne than in healthy controls [7]. A study investigating the diets of adult women in adolescence revealed a correlation between the consumption of milk and the prevalence of acne. This relationship was particularly evident in the case of semi‐skimmed milk [8]. However, acne has not been reported to be associated with fermented milk products such as yogurt and kefir [9]. The mean number of individuals consuming fermented milk products and the mean amount of fermented milk product consumed were similar between the acne and control groups in our study. The association between acne and milk, but not fermented milk products, may be attributed to the ability of probiotic bacteria, particularly lactobacilli, to reduce the levels of IGF‐1 during fermentation when they are added to milk [2]. Additionally, fermented dairy products may possess anti‐inflammatory, sebum‐reducing, and antimicrobial properties, which could be attributed to the presence of lactoferrin and Lactobacillus bacteria [10]. The lack of a significant difference in mean blood zonulin levels between the acne and control groups in those consuming > 1 glass/day of fermented milk products supports the hypothesis that fermented milk products may exert a protective effect on the intestinal barrier by increasing intestinal bacterial diversity.

In the present study, the number of individuals who consumed more than one serving per day of fast food, ready‐to‐eat desserts, more than two glasses per day of sugary soft drinks, more than one serving per day of refined cereals, and the mean amount of ready‐to‐eat desserts and refined cereals was significantly higher in the acne group. In the literature, it has been reported that patients with acne consume greater quantities of sugary soft drinks, fast food, and sweet snacks, as well as other foods with a high glycemic load and carbohydrates. Furthermore, there was a positive correlation between carbohydrate consumption and the severity of acne. A diet with a low glycemic index has been demonstrated to reduce free androgen levels by increasing the concentration of SHBG and to decrease IGF‐1 levels by increasing the concentration of insulin‐like growth factor binding protein 3 (IGFBP3) [6]. In the acne group, individuals who consumed fast food, ready‐to‐eat desserts, and sugary soft drinks had significantly higher mean blood zonulin levels than those in the control group who consumed the same foods in our study. A review of the literature revealed that blood zonulin levels increase in conjunction with total calorie and carbohydrate intake. Conversely, a reduction in blood zonulin levels and intestinal permeability has been observed in low‐carbohydrate dieters [3]. Our findings suggest a potential association between a diet with a high glycemic index and the pathogenesis of acne, possibly via increased intestinal permeability.

The relationship between chocolate consumption and acne development has been discussed in numerous publications [6]. The mean consumption of chocolate was significantly higher in patients with acne than in healthy controls in the present study. Furthermore, the number of individuals consuming milk chocolate among acne patients and the number of individuals consuming dark chocolate among the control group were significantly higher. In accordance with our findings, individuals with acne consumed greater quantities of milk chocolate and lower quantities of dark chocolate than those without acne [7]. Additionally, the mean blood zonulin level of milk chocolate consumers in the acne group was significantly higher than that of controls in our study. However, no significant difference was observed in the mean blood zonulin level of dark chocolate consumers between the two groups. The significantly elevated mean blood zonulin level observed in patients with acne who consumed milk chocolate may be attributed to its high glycemic index, largely due to its elevated sugar and milk content. This, in turn, has been demonstrated to increase intestinal permeability. In contrast, dark chocolate has been demonstrated to exhibit prebiotic effects and enhance the diversity and abundance of intestinal bacteria, and the polyphenols present in its composition have been shown to diminish intestinal permeability [11]. Furthermore, the low sugar and milk content of dark chocolate, coupled with its high cocoa concentration, has been shown to suppress TNF‐α production through its tryptophan content [12]. This may confer a protective effect against acne by reducing inflammation.

It is well documented that a diet of Mediterranean origin offers protection against acne. Patients with acne demonstrate a reduced level of adherence to the Mediterranean diet compared with control subjects. Furthermore, studies have demonstrated that individuals with acne who do not adhere to the Mediterranean diet exhibit significantly elevated levels of IGF‐1 compared with those who adhere to this dietary pattern [13]. In our study, the number of patients with acne who consumed more than one tablespoon of olive oil per day was lower than that in the control group (*p* = 0.026). Additionally, the mean amount of olive oil consumed was lower in the acne group than in the control group (*p* = 0.0001). The lack of a significant difference in mean blood zonulin levels between olive oil consumers in both groups may be attributed to the ability of olive oil and its phenolic components to regulate the intestinal microbiota and reduce intestinal permeability [14].

Although previous studies have demonstrated a protective effect of vegetable consumption on acne and a negative association between a vegetable‐poor diet and acne [15], our findings indicate that the number of individuals consuming > 1 serving/day of vegetables and acne severity was similar between the acne group and the control group. No statistically significant difference was detected between the mean blood zonulin levels of acne patients who consumed vegetables and those of the control group. These results may be explained by the fact that the individuals who participated in our study were mostly university students, and it was relatively more difficult for them to access home‐cooked meals. Additionally, the consumption of fruit has been linked to a reduced risk of acne [16]. In accordance with the literature, the number of individuals consuming fruit was significantly lower in the acne group. Furthermore, the mean blood zonulin levels of individuals who consumed fruits were similar between acne patients and controls. It has been proposed that the anti‐acne properties of fruit are attributable to its anti‐inflammatory and antioxidant effects [17].

Omega‐3 can mitigate follicular occlusion, sebum production, and inflammation by inhibiting the synthesis of IGF‐1 and leukotriene B4, which are both implicated in inflammatory processes [18]. In studies examining the correlation between fish, a food source rich in omega‐3 fatty acids, and acne, patients with acne exhibited decreased consumption of fish. Consequently, it is recommended that fish be added to the diet of patients with acne [7, 15]. In our study, the number of individuals consuming fish and the amount of fish consumed in the acne group were markedly lower than those in the control group. Furthermore, no notable discrepancy was identified between the mean blood zonulin levels of fish‐consuming acne patients and controls.

The relationship between eggs and acne has been the subject of only a limited number of studies. Although some studies indicate that individuals with acne consume more eggs and that the incidence of acne may increase following egg consumption [6], other publications suggest that eggs may have a protective effect against acne due to their high vitamin A and vitamin D content [10]. In the present study, the low number and amount of egg consumption in the acne group, in conjunction with the higher mean blood zonulin level compared to the control group, may be explained by the fact that eggs are a rich source of vitamins A, D, and selenium. It is established that vitamin A increases the expression of zo‐1 and zo‐2, which are tight junction proteins in the intestine. Additionally, vitamin D has been demonstrated to regulate the intestinal barrier epithelium by decreasing blood zonulin levels [19]. Additionally, selenium, which is plentiful in eggs, is believed to enhance the gastrointestinal mucosal barrier and regulate intestinal permeability by mitigating oxidative stress [20].

A number of studies have indicated a correlation between the consumption of meat and the development of acne lesions. Furthermore, studies have demonstrated that individuals with acne tend to consume more red meat [7]. In our study, the number of individuals consuming red meat and the mean consumption amount were higher in the acne group (*p* values 0.001 and 0.0001, respectively). Red meat is a rich source of branched‐chain amino acids, including leucine. There is evidence that leucine may contribute to the development of acne by increasing lipogenesis in sebaceous glands [21]. The mean blood zonulin level of acne patients who consumed red meat was also elevated (*p* = 0.020), and as red meat consumption increased, blood zonulin levels also increased in the acne group (*p* = 0.018). The microbiota of mice fed a diet rich in red meat was similar to the microbiota of patients with ulcerative colitis and celiac disease, and the intestinal integrity of these mice was impaired [22]. In conclusion, the consumption of red meat may affect zonulin levels and, consequently, trigger acne by disrupting the intestinal microbiota and permeability. The mean number of individuals consuming more than two servings per day of processed meat and the mean amount of processed meat consumed were significantly higher in patients with acne (*p* values of 0.007 and 0.040, respectively), and the mean blood zonulin level was markedly elevated in these patients (*p* value: 0.033). The consumption of processed meat may result in a reduction in *Bifidobacteria spp*. within the gut microbiota, which may subsequently lead to an increase in intestinal permeability [23].

The mean amount of legume consumed and the number of individuals consuming more than 100 g of legumes per week were higher in the acne group than in the control group (*p* values of 0.019 and 0.030, respectively). In terms of the relationship between acne severity and legume consumption, there was significantly higher mean legume consumption in the severe acne group (*p* = 0.038). In an experiment conducted on rats, it was demonstrated that lectin, which is highly concentrated in legumes, increased intestinal permeability [24]. Our findings are consistent with those of previous studies and demonstrated that the mean blood zonulin levels were markedly elevated in legume consumers relative to controls within the acne group. It is possible that there is an association between legume consumption and acne, as well as acne severity, due to an increase in intestinal permeability. In our study, the number of individuals consuming nuts and the mean amount of nut consumption were significantly higher in the control group (*p* = 0.015). The mean blood zonulin level in the acne group was higher than in the control group among those who consumed more than one serving of nuts per day (*p* = 0.004). It has been previously documented in the literature that nuts are a rich source of polyphenols (especially tannins), short‐chain fatty acids (such as butyrate) and fiber. Furthermore, nuts positively regulate the intestinal microbiota. Butyrate, in particular, has been demonstrated to reduce intestinal permeability [25]. Our findings are consistent with those of previous studies, and it can be postulated that nut consumption has a protective effect against acne by decreasing intestinal permeability.

The strengths of our study are that the sociocultural and sociodemographic characteristics of the patient and control groups were compatible with each other, comprehensive dietary questioning was performed in detail by the physician conducting the study, not by the interviewer, and this is the first study in the literature on zonulin and intestinal permeability in the pathogenesis of acne vulgaris. One of the limitations of our study is the high number of patients with moderate acne because our hospital is a tertiary healthcare institution. Additionally, the number of patients was restricted due to the lengthy and comprehensive nature of the nutrition questionnaire.

The results of our study suggest that increased intestinal permeability may contribute to the pathogenesis of acne. Additionally, our findings suggest that certain foods, including red meat, milk chocolate, low‐fat milk, fast food, and ready‐to‐eat desserts, may have a negative effect on zonulin levels. However, it is important to note that these effects are complex and require further investigation. It may be recommended that foods such as fish, olive oil, vegetables and fruits, eggs, and nuts be included in an acne‐protective diet. Furthermore, larger‐scale studies are needed to examine the relationship between diet and acne and zonulin levels.

## Author Contributions

R.C.G. and M.Y. designed the study. D.K.D. made biochemical measurements on the blood samples. R.C.G. and İ.E. analyzed the data. S.K. performed the analytic calculations. R.C.G. and M.Y. wrote the paper. M.Y., İ.E., and S.K. conceived the study and were in charge of overall direction and planning. All authors have read and approved the final manuscript.

## Ethics Statement

In accordance with research and publication ethics, ethical approval was obtained for this study with the approval of the Süleyman Demirel University Faculty of Medicine Chief Physician and Ethics Committee dated 27.10.2022 and numbered 72867572‐050.01.04‐386516.

## Conflicts of Interest

The authors declare no conflicts of interest.

## Data Availability

The data that support the findings of this study are available from the corresponding author upon reasonable request.
